# Globalisierung in der Krise?

**DOI:** 10.1007/s10273-021-3039-6

**Published:** 2021-11-18

**Authors:** ZG Überblick

**Keywords:** F10, F13, F60

## Abstract

Die Globalisierung hat im vergangenen Jahrzehnt deutlich an Geschwindigkeit verloren. Der Welthandel wuchs in den 2010er Jahren langsamer als die Weltproduktion. Protektionistische Tendenzen bremsen den Welthandel, auch wenn der Multilateralismus nach der Präsidentschaft Donald Trumps wieder an Rückhalt gewonnen hat. Auch die Corona-Pandemie und Lieferengpässe haben Sand in das Getriebe der Globalisierung gestreut. Lediglich der internationale Handel mit Dienstleistungen zeigt noch ein robustes Wachstum. Deutschlands enge wirtschaftliche Verflechtung mit der Welt bedeutet auch, dass Störungen in Lieferketten oder Nachfrageeinbrüche während einer Pandemie äußerst negativ zu bewerten sind und geeignete Strategien für die globale Gesundheitssicherheit verfolgt werden sollten.

## Abstract (en)

Globalisation has lost considerable speed in the last decade. World trade grew more slowly than world production in the 2010s. Protectionist tendencies are slowing down world trade, even if multilateralism has regained support in the post-Trump presidency era. The coronavirus pandemic and supply bottlenecks have also thrown sand into the gears of globalisation. Only international trade in services is still showing robust growth. Germany’s close economic ties with the world also mean that disruptions in supply chains or slumps in demand during pandemics are to be viewed extremely negatively and appropriate strategies for global health security should be pursued.

